# Outcome of intracerebral cavernoma treated by Gamma Knife radiosurgery based on a double-blind assessment of treatment indication

**DOI:** 10.1186/s13014-021-01885-4

**Published:** 2021-08-28

**Authors:** Chiung-Chyi Shen, Ming Hsi Sun, Meng-Yin Yang, Weir-Chiang You, Meei-Ling Sheu, Yen-Ju Chen, Ying Ju Chen, Jason Sheehan, Hung-Chuan Pan

**Affiliations:** 1 Department of Neurosurgery, Taichung Veterans General Hospital, Taichung, Taiwan; 2 Basic Medical Education Center, Central Taiwan University of Science and Technology, Taichung, Taiwan; 3 Department of Radiation Oncology, Taichung Veterans General Hospital, Taichung, Taiwan; 4 Institute of Biomedical Science, National Chung-Hsin University, Taichung, Taiwan; 5 College of Humanities and Social Sciences, Providence University, Taichung, Taiwan; 6 Department of Neurosurgery, University of Virginia, Charlottesville, VA USA; 7 Department of Medical Research and Neurosurgery, Taichung Veterans General Hospital, 1650 Taiwan Boulevard Sec.4, Taichung, 40705 Taiwan; 8 Rong Hsing Research Center for Translational Medicine, National Chung Hsing University, Taichung, Taiwan

## Abstract

**Background:**

The benefit and the risk profile of Gamma Knife radiosurgery (GKRS) for intracerebral cavernoma remains incompletely defined in part due to the natural history of low incidence of bleeding and spontaneous regression of this vascular malformation. In this study, we retrieved cases from a prospectively collected database to assess the outcome of intracerebral cavernoma treated with GKRS using a double blinded review process for treatment.

**Methods:**

From 2003 to 2018, there were 94 cases of cavernoma treated by GKRS in the doubly blinded assessments by two experienced neurological and approved for GKRS treatment. All the patients received GKRS with margin dose of 11–12 (Gray) Gy and afterwards were assessed for neurological outcome, radiologic response, and quality of life.

**Results:**

The median age of the patients was 48 (15–85) years with median follow up of 77 (26–180) months post SRS. The mean target volume was 1.93 ± 3.45 cc. In those who has pre-SRS epilepsy, 7 of 16 (43.7%) achieved seizure freedom (Engel I/II) and 9 of 16 (56.3%) achieved decreased seizures (Engel III) after SRS. Rebleeding occurred in 2 cases (2.1%) at 13 and 52 months post SRS. The radiologic assessment demonstrated 20 (21.3%) cases of decreased cavernoma volume, 69 (73.4%) were stable, and 5 (7.3%) increased size. Eighty-seven of 94 (92.5%) cases at the last follow up achieve improvement in their quality of life, but 7 cases (7.4%) showed a deterioration. In statistical analysis, the effective seizure control class (Engel I/II) was highly correlated with patient harboring a single lesion (*p* < 0.05) and deep seated location of the cavernoma (*p* < 0.01). New neurological deficits were highly correlated with decreased mental (*p* < 0.001) and physical (*p* < 0.05) components of quality of life testing, KPS (*p* < 0.001), deep seated location (*p* < 0.01), and increased nidus volume (*p* < 0.05). Quality of life deterioration either in physical component (*p* < 0.01), mental component (*p* < 0.01), and KPS (*p* < 0.05) was highly correlated with increased cavernoma volume.

**Conclusion:**

Low margin dose GKRS for intracerebral cavernoma offers reasonable seizure control and improved quality of life while conferring a low risk of treatment complications including adverse radiation effect.

**Supplementary Information:**

The online version contains supplementary material available at 10.1186/s13014-021-01885-4.

## Background

Intracerebral cavernoma (CM) are uncommon in the general population, with a prevalence ranging from 0.3 to 0.6% based on large autopsy series and prospective cohort studies. The increasing incidence of cavernoma is largely due to diagnostic advances with widespread use of magnetic resonance imaging (MRI) in clinical practice (prevalence 0.4–0.9%) [1–4]. Individuals with CMs can present with seizures (23–50% of cases), headaches (6–52%), focal neurological deficits (20–45%), or hemorrhages (9–56%) [1, 5–10]. The extent of permanent neurological deficits highly correlates with the number of recurrent hemorrhages, and re-bleeding episodes tend to occur at progressively shorter time intervals [11]. In patients with a symptomatic cavernoma, microsurgery is the best treatment for CM, especially given advances in microsurgical techniques and neuronavigation-guided approaches [12]. For patients with deeper seated or eloquently situated cavernomas, Gamma Knife radiosurgery (GKRS) is considered as an alternative [13–15].

The use of radiosurgery for cavernoma remains controversial especially for the primary goal of reducing the bleeding rate. Some authors have favored radiosurgery for intracranial cavernoma, due to a reduced risk of hemorrhages after a latency period of 2–3 years [16–18]. But others are less convinced about the benefits of SRS for cavernomas for a variety of reasons. First, the hemorrhage rate, particularly for retrospective series, is not simple to calculate due to the appearance of cavernoma in de novo and referral and treatment biases [19]. Second, the high-risk CM patients are usually selected to undergo surgery after SRS and thus deflate the post-SRS hemorrhage rates with time [20]. Ironically, in some reports, SRS itself can also induce de novo CM development [21–23]. Furthermore, the risk of a CM rebleeding is typically high for 2–3 years after the initial hemorrhage and, thereafter, cavernoma re-hemorrhage after SRS appears to be reduced after this period of time [24]. The temporal clustering of hemorrhagic events might give a false impression of how aggressive a lesion will be in the long term. The decline in hemorrhagic events observed after treating CMs with SRS could, therefore, be a reflection of the natural history of the lesions rather than the result of radiosurgery [20, 25, 26]. Finally, cavernous malformations are dynamic lesions that may exhibit enlargement, regression, or even de novo formation [10, 27, 28]. Hence, the beneficial effect of SRS in altering the natural history of cavernoma continues to be questioned.

The risk of seizures was estimated to be 1.34% per person-year for solitary CMs and 2.48% per person-year for multiple lesions [29]. The assessment of gamma knife on the seizure control rate based on the different study design achieved the seizure free rate from 31 to 53% and decreased seizure frequency from 45 to 66%, but without any treatment-related death [14, 18, 30–32]. Thus, it seems that GKRS seems to be a rational approach for improving seizure frequency associated with a cavernoma.

The outcome of radiosurgery on the intracerebral cavernoma remains controversial. One way to verify the actual effect of GKRS is by clinical observation during a longer follow-up period. In addition, one could study the effects of GKRS on quality of life and seizures in cavernoma treatment patients. In this study, we prospectively evaluated the outcomes of GKRS in cavernoma patients who were *double-blind assessment* by two independent neurosurgeons.

## Methods

### Patient population

From 2003 to 2018, there were 121 cases of intracranial cavernoma blindly approved by the two independent neurosurgeons excluding the in-charge neurosurgeon for the GKRS based on the patients’ medical records and imaging findings at the Central Bureau of Health Insurance, Taichung, Taiwan, to determine whether GKRS was the appropriate treatment. The approval criteria was based on the consensus of Taiwan Neurosurgical Society on for GKRS including one or more of the following: recurrence of cavernoma after craniotomy, *target* volume less than 20 cc or maximum diameter less than 3.5 cm, vulnerable location for the nidus removal, severe illness inappropriate for general anesthesia, or KPS > 70. Finally, there were 105 of 121 (86.7%) cases approved for Gamma Knife treatment. There were 11 cases lost to follow up, and, as such, 94 (89.5%) cases were included in this study. The treatment protocol was presented as a schematic flowchart shown in Additional file 1: Figure S1. The study was approved by the ethical committee of Taichung Veterans General Hospital on record No. CE21185B.

### Radiosurgical technique

After the patient had received a local anesthetic agent, the Leksell G head frame was affixed to the head, and the patient was monitored for blood pressure, oxygenation, and electrocardiography. All patients were treated with a Leksell Gamma Knife model D (Elekta AB) by a team consisting of a neurosurgeon, neuroradiologist, radiation oncology, and medical physicist. All patients underwent GKRS with low margin dosage of 11–12 Gy prescribed to the *target* at the isodose line of 50–60% with radiation dose constrains with optic apparatus < 8 Gy, brain stem < 12 Gy, and lens < 2 Gy. Radiosurgery dose plans, with single or multiple isocenters, were created, and the targeted margin of the cavernoma was considered to be the region characterized by mixed signal change within the T2-weighted signal-defined hemosiderin ring [33].

### Imaging technique

The target lesions were typically imaged using a 1.5-T MR imaging unit (GE Medical Systems). Target localization was performed using T1-weighted, fast-spin-echo T2-weighted, spoiled-gradient recalled, and time-off light imaging. Additional T1-weighted, spoiled-gradient recalled, and time-of-flight sequences were also obtained after administration of gadolinium (Gd). The axial volume acquisition of 256 × 256 matrices was divided into 1-mm thickness without a gap. All patients gave informed consent to receive a Gd injection in accordance with Taiwan guidelines concerning Gd administration during MR imaging examinations.

### Clinical follow up and assessment of Life quality

The patients received regular follow up at 3–6 month intervals after GKRS including neurological examination and record of frequency, intensity and drug dosage in patients with a seizure history. SF-36 is a well-validated instrument for measuring quality of life (QOL) [34]. It covers 8 domains including physical function (PF), role limitation due to a physical problem (RP), bodily pain (BP), general health (GH), vitality, social functioning (SF), role limitation due to an emotional problem (RE), and mental health (MH). In this study, BP was specifically limited to headache and facial pain, and these were clearly described for the participants. In general, the physical component summary covered PF, RP, BP, and GH, whereas the mental component summary included vitality, SF, RE, and MH. Scores on the SF-36 scale range from 0 to 100, with higher scores indicating better condition. The QOL data were collected prior to GKRS and at last out-patient follow-up. The Karnofsky Performance Score (KPS) spans from 100 to 0, where 100 is “perfect” health and 0 is death [35]. KPS was also collected by the clinical team before GKRS and at last follow up.

### Imaging follow-up

All patients underwent routine MR imaging examinations 6–12 months after GKRS. More specifically, T1-weighted images were obtained with or without administration of Gd, and T2-weighted images were obtained to evaluate whether there were any adverse treatment effects. If patients experienced new neurological deficits (increased seizure frequency, impairment sensory of motor function), they underwent additional imaging examinations at the time of newly neurological deficits to evaluate for radiologic changes associated with these clinical changes. The assessment of volume alteration was based on our previous investigation with volume enlargement by 20% defined as increase, volume reduction by 20% defined as decrease, and volume changes of less than 20% from baseline defined as stable [36].

### Statistical analysis

Descriptive statistics were computed using standard methods to calculate mean ± standard deviation or median values with ranges. Factors contributing to seizure frequency, imaging alteration, neurological outcome, and quality of life that were assessed by the Mann–Whitney test, Chi-Square test, and Fisher’s Exact test. Cox regression analysis was used to investigate the risk factors for the new neurological deficits. Logistical regression testing was used for the assessment of the associated factors related to control of seizure and improvement in QOL. A *p* value < 0.05 was considered significant.

## Results

### Patient demographics and treatment parameters

The median age of the patients was 48 year old at the time of GKRS, and there was a male/female ratio of 55–39. The clinical diagnosis included 78 cases with hemorrhage and 16 with seizure. Forty one cases presented with pre-existing neurological deficits included motor weakness of 21, sensory impairment of 15, and gait imbalance of 5. Cavernoma locations include 20 cases in the brainstem, 36 in deep seated location and 38 in the sub-cortical region. The mean treated volume was 1.93 ± 3.45 cc. The median margin dose was 12 Gy (11–12). The scores of pre-GKRS KPS were 66.1 ± 7.4. Pre GKRS scores of SF-36 included general health (34.4 ± 15.2), pain (headache) (34.5 ± 21.2), social function (25.5 ± 16.6), emotional well being (35.6 ± 16.9), energy/fatigue (34.2 ± 14.2), role limitation by emotional health (15.6 ± 18.1), role limitation by physical health (28.7 ± 19.1), physical role (33.4 ± 9.4) (Table 1). *Patients’ treatment indication* stratified by the pre-GKRS indications of intracranial bleeding (ICH) and seizure are shown in Table 2.

**Table 1:** The characteristics of the patients (n = 94)

Age (years)	46.39 ± 15.8	
Sex		
Female	55	(58.51%)
Male	39	(41.49%)
Treatment indication		
ICH/craniotomy	78/6	(82.98%/6.3%)
Seizure	16	(17.02%)
Interval from diagnosis to GKRS (months)	4.94 ± 5.2	
Pre-existing neurological deficits	41	(43.62%)
Familial history	6	(6.38%)
Multiple lesions	28	(29.79%)
Location		
Brain stem	20	(21.28%)
Deep seated	36	(38.30%)
Subcortex	38	(40.43%)
Venous abnormality	15	(15.96%)
Target volume (TV) (cc)	1.93 ± 3.45	
Margin dose (Gray)	11.6 ± 1.1	
Physical component	131.01 ± 34.30	
Mental component	118.04 ± 48.29	
KPS	66.06 ± 7.36	

Continuous data were expressed mean ± SD

Categorical data were expressed number and percentage

*ICH* intracerebral hemorrhage

**Table 2:** The characteristics of the patients stratified by treatment indication

	ICH(n = 78)	Seizure (n = 16)	*p* values
Age	47.53 ± 15.19	40.88 ± 18.01	0.146
Sex			
Female	44	11	0.526
Male	34	5	
Craniotomy	3	3	0.06
Interval from diagnosis to GKRS (months)	4.51 ± 4.91	7.0 ± 6.18	0.038
Pre-existing neurological deficits	41	0	< 0.001
Familial Hx	3	3	0.059
Multiple lesions	21	7	0.231
Location			
Brain stem	20	0	0.017
Deep seated	31	5	
Subcortex	27	11	
Venous abnormality	14	1	0.454
Target volume (TV)	1.89 ± 3.64	2.11 ± 2.41	0.577
Margin dose (Gray)	12 (11–12)	12 (11–12)	0.392
Physical component	126.12 ± 33.60	154.84 ± 27.73	0.001
Mental component	345.08 ± 77.92	382.50 ± 12.45	0.001
KPS	64.97 ± 7.34	71.88 ± 4.03	0.01

Mann–Whitney test. ^c^Chi-Square test. Fisher’s Exact test. *p < 0.05; **p < 0.01

Continuous data were expressed mean ± SD

Categorical data were expressed number and percentage

*ICH* intracerebral hemorrhage

### Clinical and imaging outcome

**Fig. 1:**
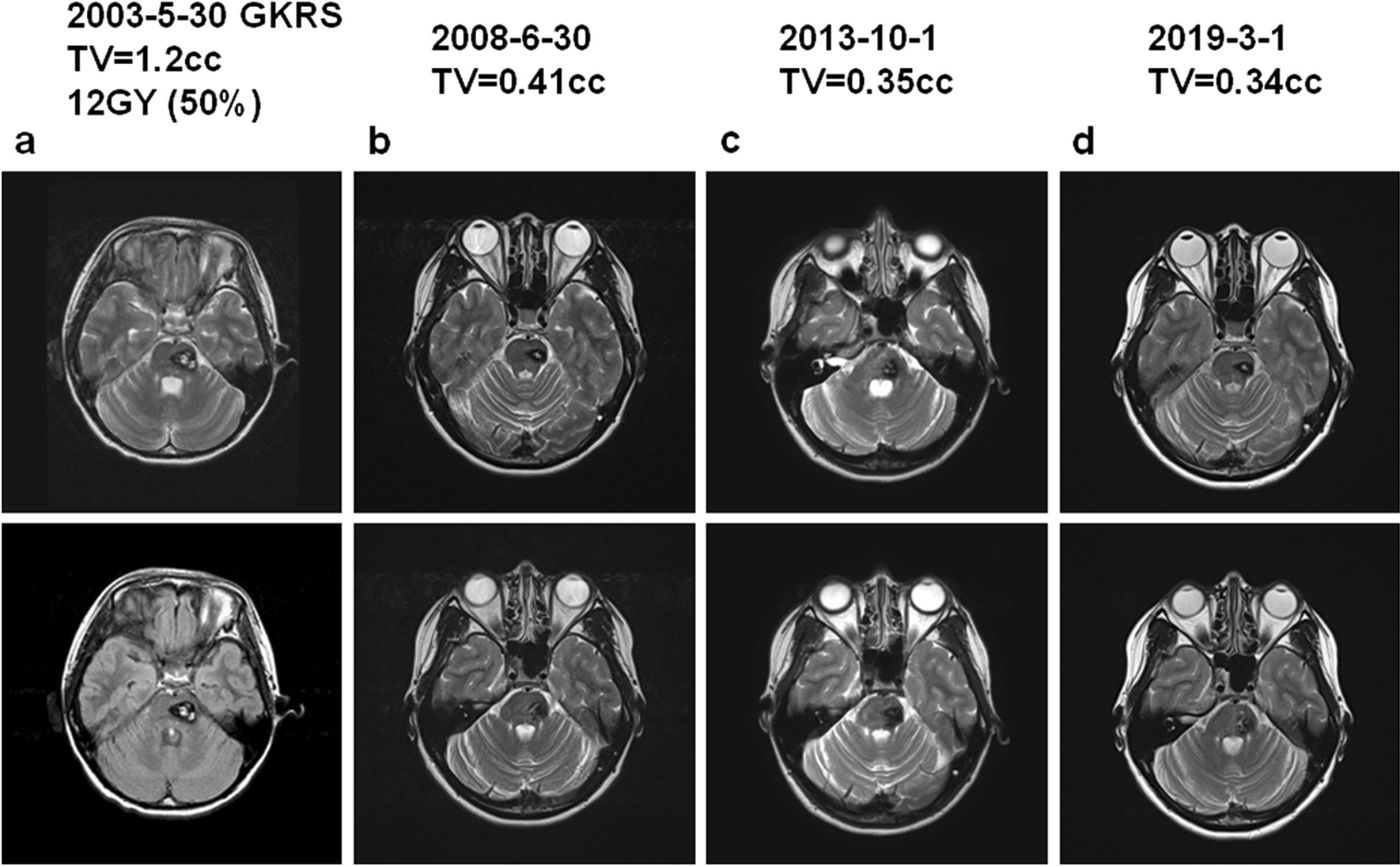
Cavernoma treated with GKRS with decreased size. A 13 year old girl suffered right side limb weakness with muscle power of grade IV and received GKRS with the regression of nidus. **a** MRI imaging of T2, Flair at the time of GKRS with radiation volume of 1.2 cc with 12 Gy in 50% line, **b** MRI imaging of T2 and Flair 6 years after GKRS with nidus volume of 0.41 cc, **c** MRI imaging of T2 and Flair 10 years after GKRS with nidus volume of 0.35 cc and **d** MRI imaging of T2 and Flair 16 years after GKRS with nidus volume of 0.34 cc

**Fig. 2:**
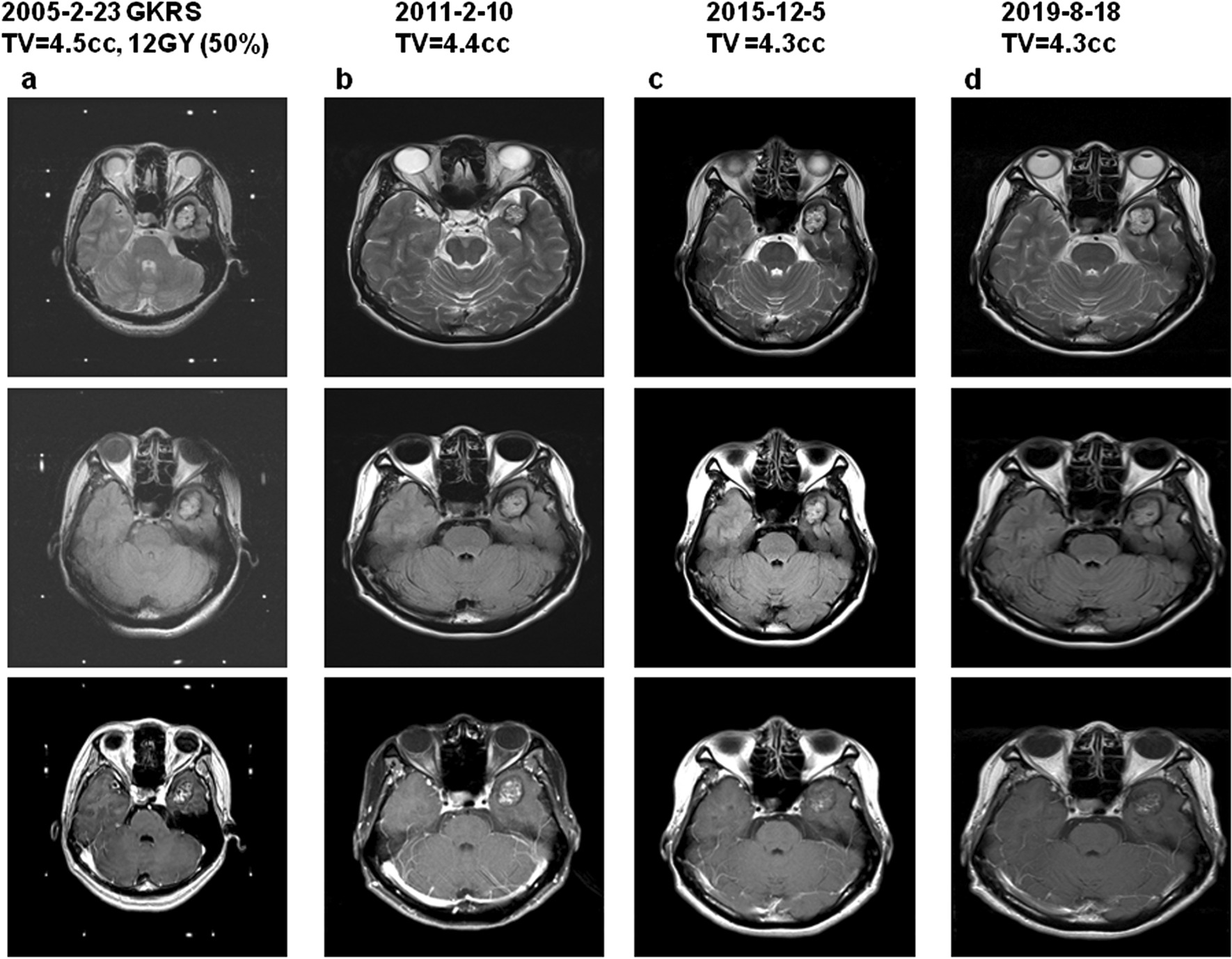
Cavernoma treated with GKRS and achieving stable size. A 30 year old female presented with tonic–clonic seizure treated with Gamma Knife radiosurgery with stable size of nidus and seizure control of Engel II. **a** MRI imaging of T2, Flair and T1 with contrast at the time of GKRS with radiation volume of 4.5 cc with 12 Gy in 50% line. **b** MRI imaging of T2, Flair and T1 with contrast 6 years after GKRS with nidus volume of 4.4 cc, **c** MRI imaging of T2, Flair and T1 with contrast 10 years after gamma knife treatment with nidus volume of 4.3 cc and **d** MRI imaging of T2, Flair and T1 with contrast 14 years after GKRS with nidus volume of 4.3 cc

**Fig. 3:**
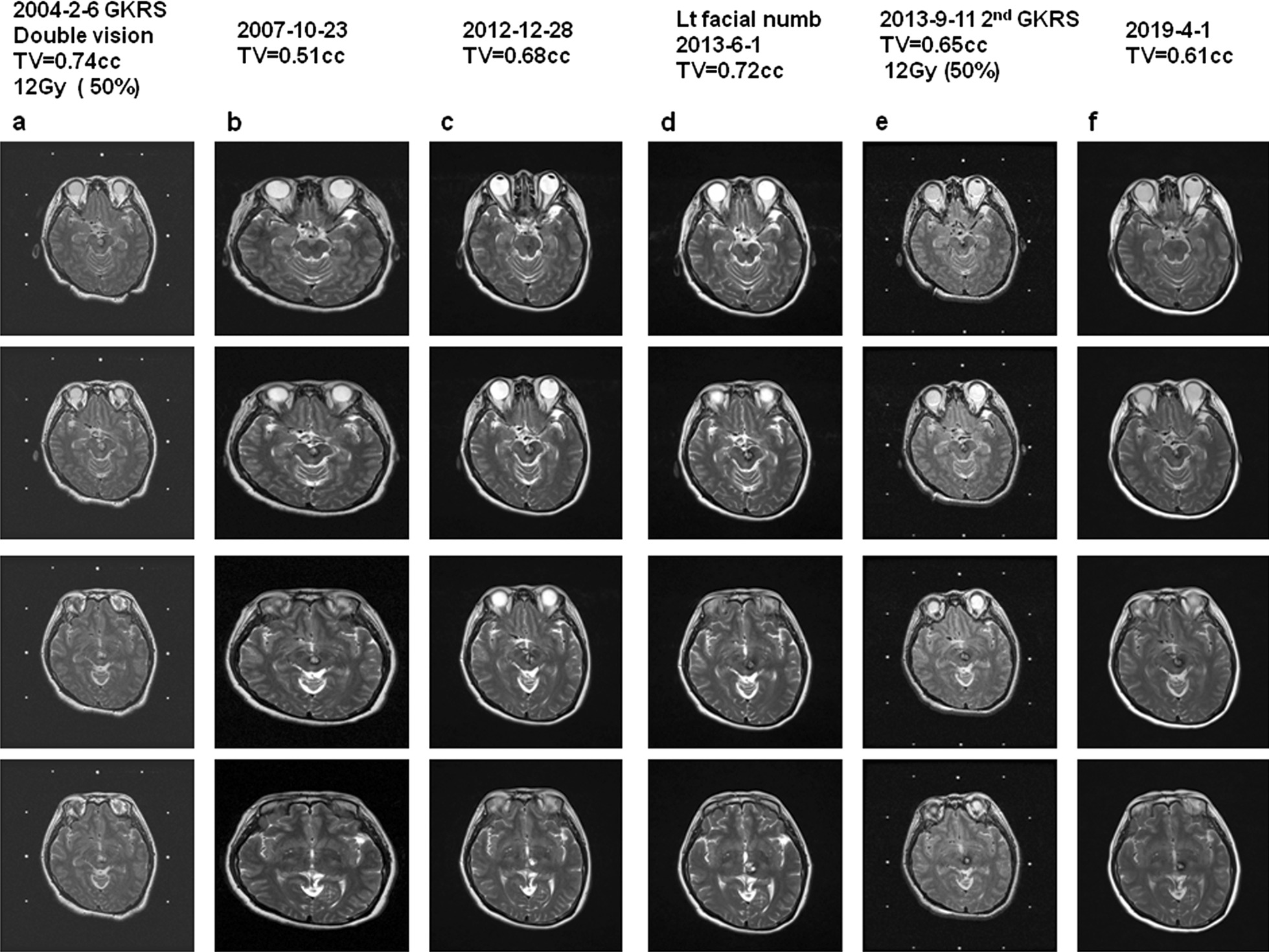
Cavernoma treated with nidus progression followed by second GKRS. A 39 year old female suffered double vision treated with GKRS and underwent a second GKRS due to increased volume of the nidus with associated symptom of facial numbness **a** MRI imaging of T2 at the time of GKRS with treatment volume of 0.74 cc with 12 Gy in 50% line, **b** MRI imaging of T2 3 years after GKRS with nidus volume of 0.51 cc, **c** MRI imaging of T2 weighted 8 years after GKRS with nidus volume of 0.68 cc, **d** MRI imaging of T2 9 years after GKRS with nidus volume of 0.72 cc, **e** MRI imaging of T2 at the time of the second GKRS with radiation volume of 0.65 cc with 12 Gy in 50% line and **f **MRI imaging of T2 15 years after GKRS with a nidus volume of 0.61 cc

The median follow up period was 77 months. The imaging analysis demonstrated 20 (21.3%) cases of decreased nidus (Fig. 1), 67(73.4%) stable (Fig. 2), 5 (7.3%) with increased size (Fig. 3 and Additional file 1: Figure S2), and 2 cases with rebleeding (Additional file 1: Figure S3).

Mean post-GKRS KPS were 92.8 ± 11.4. Post GK scores of SF-36 included general health (88.8 ± 18.1), pain (headache) (88.7 ± 20.6), social function (84.9 ± 20.2), emotional well bening (88.5 ± 17.9), energy/fatigue (86.2 ± 19.3), role limitation by emotional health (80.5 ± 24.2), role limitation by physical health (87.2 ± 24.8), physical role (86.7 ± 17.4). There were significant differences of life quality after GKRS in all patients (Additional file 1: Figure S3a) and also in those stratified by pre-GKRS indications of cavernoma hemorrhage and seizures (Additional file 1: Figure S3b).

Following GKRS, seven cases demonstrated new neurological deficits including 2 cases (2.1%) with re-bleeding at time point of 13 and 52 months following radiosurgery and 5 cases with deficits associated with increased nidus volume at time point of 24, 52, 80, 96, 108, and 134 months, respectively. The *associated* factors for new neurological deficits were shown in Table 3 including brainstem location (*p* < 0.01), venous abnormality (*p* < 0.01), nidus increased after GKRS (*p* < 0.001), post GKRS KPS (*p* < 0.01), post GKRS physical component (*p* < 0.05), and post GKRS mental component (*p* < 0.01). The risk factors for the new neurological deficits consisted of pre-GKRS physical component (*p* < 0.05), pre-GKRS mental component (*p* < 0.05) and venous abnormality (*p* < 0.05) in Table 4. Those patients who developed new neurological deficits also showed no improvement in life quality as illustrated in Additional file 1: Figure S4.

**Table 3:** Characteristics of the patients with new neurological deficits

	No (n = 87)	Yes (n = 7)	*p* value
Age	46.8 ± 15.8	40.8 ± 15.8	0.376
Sex ration (F/M)	1.35	2.25	0.695
ICH history	71	7	0.599
Seizure history	16	0	0.512
Time to GKRS (months)	4.95 ± 5.23	4.86 ± 5.11	0.913
Pre-existing neurological deficits	37(42.5%)	4(57.5%)	0.695
TV (cc)	2.0 ± 3.56	0.98 ± 1.29	0.264
Familial Hx	6	0	1
Multiple lesion	27	1	0.670
Brain stem Location	15 (17.2%)	5 (71.4%)	*p* < 0.01
Venous abnormality	11 (12.64%)	4 (57.15%)	*p* < 0.01
Nidus increased post GKRS	1 (1.14%)	4 (87.1%)	*p* < 0.001
Post-GKRS KPS	95.40 ± 5.67	60.0 ± 14.14	*p* < 0.01
Post GKRS Physical component	113.38 ± 47.08	79.3 ± 45.79	*p* < 0.05
Post GKRS Mental component	357.14 ± 36.01	129.30 ± 95.67	*p* < 0.001

Mann–Whitney test. ^c^Chi-Square test. Fisher’s Exact test. *p < 0.05; **p < 0.01

Continuous data were expressed mean ± SD

Categorical data were expressed number and percentage

*ICH* intracerebral hemorrhage

**Table 4:** Risk factors for the new neurological deficits

	Simple model		Multiple model	
	HR (95%CI)	*p* value	HR (95%CI)	*p* value
Age	1.00 (0.95–1.04)	0.909		
Sex				
Female	ref			
Male	0.73 (0.13–4.07)	0.715		
Time from diagnosis to GK (months)	0.95 (0.78–1.14)	0.565		
Neurological deficits	3.29 (0.58–18.61)	0.179		
TV (cc)	0.91 (0.58–1.43)	0.693		
Margin dose (Gy)	1.33 (0.67–2.63)	0.419		
Multiple lesions at GK	0.63 (0.07–5.62)	0.678		
Physical component	0.95 (0.92–0.99)	0.019	0.97 (0.92–1.01)	0.128
Mental component	0.97 (0.95–0.996)	0.019	0.98 (0.96–1.01)	0.125
Location				
Brain stem + deep seated	ref			
Subcortical	0.33 (0.04–2.89)	0.319		
Venous abnormality	5.36 (1.02–28.06)	0.047	3.03 (0.59–15.46)	0.183

Cox regression. *p < 0.05; **p < 0.01

*HR* hazard ratio

In 16 cases with pre GKRS seizure, 7 cases reach the Engel I–II and 9 cases of Engel III at last follow up. There was no case of Engel IV. There were significantly different parameters for effective seizure control (Engel I/II) shown in Table 5 including post GK mental component (*p* < 0.004) and a single lesion (*p* < 0.05). In logistic regression analysis, only the single lesion showed the favorable effect (*p* < 0.056).

**Table 5:** Characteristics of the patients in the seizure control

	Engel 1–2 (n = 7)	Engel 3 (n = 9)	*p* value
Age	49.86 ± 20.23	33.89 ± 13.27	0.119
Sex (F/M)	5/2	6/3	1
History of craniotomy	1	2	1
Interval from diagnosis to GKRS	8.00 ± 8.77	6.22 ± 3.49	0.898
Physical component at GKRS	162.14 ± 28.15	149.17 ± 27.64	0.303
Mental component at GKRS	384.29 ± 13.28	381.11 ± 12.38	0.639
KPS at GKRS	72.86 ± 4.88	71.11 ± 3.33	0.55
Physical component after GKRS	141.27 ± 18.94	142.76 ± 30.83	0.938
Mental component after GKRS	397.29 ± 3.86	377.59 ± 15.43	0.004
KPS after GKRS	98.57 ± 3.78	97.78 ± 4.41	1
TV (cc)	1.83 ± 2.04	2.33 ± 2.76	0.123
Multiple lesions	1	6	0.04
Location (subcortical region)	3	8	0.106
Post GKRS volume (increase)	0	1	0.652

Mann–Whitney test. ^c^Chi-Square test. Fisher’s Exact test. *p < 0.05; **p < 0.01

Continuous data were expressed mean ± SD

Categorical data were expressed number and percentage

In the logistical regression analysis, QOL deterioration either in physical component or mental component and a decline in KPS were highly correlated to increased volume of nidus (Additional file 1: Tables S1–S3).

## Discussion

The assessment of intracranial cavernoma outcomes after GKRS is confounded by the ill-defined incidence of bleeding rate, fluctuation of nidus volume, de-novo growth, and temporal hemorrhage clustering. Aside from the difficulty in the assessment of response in bleeding tendency, we found that the decreased seizure frequency and improved life quality were major contributors for the beneficial effects of GKRS on intracranial cavernoma patients.

There were some arguments in the assessment of a decreased cavernoma bleeding rate following GKRS. These debates included the longer latency to assess the rebleeding rate [16–18], selection and treatment biases [19], uneven allocation of the patients to operation [20], the temporal clustering of hemorrhage events [20, 25, 26], de novo CM development [21–23], and dynamic changes in CM [10, 27, 28]. In this study, there were only two cases experiencing hemorrhage during the follow up at 13 and 52 months post-GKRS. Due to the low incidence of bleeding and the difficulty in defining the hemorrhage episode before GKRS, the factors subjected to analysis did demonstrate significant relationships to the development of rebleeding.

QOL improvement was a useful tool for the assessment of intracranial lesions treated by the GKRS [37, 38]. In general, the SF-36, BCM-20 and KPS were used for the assessment of life quality after GKRS. In the QOL assessment, the parameters obtained should ideally be assessed in a periodic and continuous fashion [34, 35, 37, 38]. In this study, the SF-36 and KPS data were only obtained at the time point of GKRS and the last outpatient follow up. Thus, the power of the assessment was decreased.

The effect of gamma knife on cavernoma related to seizure control was various due to the different approach in the study design. In one series, seizure control following Gamma Knife was achieved in 53% of patients with Engel Grade I or II, and there was no treatment-related death [18]. In another large series including 291 patients enrolled, 31% were reported to be seizure free and 35% exhibited a decreased seizure frequency [31]. In 28 patients whose chief complaint was seizures, there was 18 (64%) patients presenting a decrease in seizure frequency, but no definite grading in seizure control [32]. In the 65 patients, seizures were controlled without anticonvulsant medication in 81.8% (Engel I) [14]. In 44 of 112 (39%) patients with seizure, 45% exhibited improvement of their seizures without mentioning the grading in seizure control [30]. Thus based upon published findings, GKRS seems to be a rationale approach for improving seizure frequency associated with a cavernoma.

There is still a debate concerning the optimal radiosurgical dose for cavernoma treatment to achieve a beneficial response and minimize side effects. Doses exceeding 15–16 Gy have previously demonstrated significant radiation edema [32, 39]. In lesions located at in the brainstem even with a margin dose of 13 Gy, there is substantial increase in radiation induced complication [40], and it seem that 13 Gy is the upper margin dose without significant risk of adverse effects for radiosurgically treated cavernomas [33]. In some anecdotal report, a margin dose of as low as 10 Gy has significant effect in cavernoma shrinkage [41]. In this study, a margin dosage of 11–12 Gy afforded recognizable nidus shrinkage or stability without appreciable adverse effect. Thus the optimal dose and threshold for radiation-related complication for CMs have not been defined until now. It seems that there is a need to explore the issue further particularly for specific sites such as the brainstem.

The effects of GKRS on intracerebral cavernoma are confounded by many factors which are difficult to control, and, therefore, the role of GKRS for cavernomas remains controversial. The only way to verify a beneficial effect of the treatment is to demonstrate no increased annual risk of re-bleeding and no appreciable complications from the treatment itself. Based on the above assumption, we applied a low margin dose of 11–12 Gy to treat the cavernoma and found that most patients demonstrated decreased seizure frequency, stabilization of the cavernoma, and improvement in QOL. Also, there were no definite adverse effects associated with GKRS. Therefore, a low margin dose of 11–12 Gy in the treatment of cavernoma seems to be a reasonable approach.

## Conclusion

Low margin dose GKRS for intracerebral cavernoma seems to be a reasonable approach which reduces seizure frequency and improves quality of life in the majority of patients. This treatment appears to be without appreciable risk of adverse radiation effects.

## Supplementary Information

**Additional file 1**. Figure S1: The treatment protocol was presented as a schematic flowchart. Figure S2: Cavernoma treated with nidus progression followed by second GKRS and craniotomy. A 29 year old female suffered facial numbness treated with GKRS and received a second GKRS due to increased volume of nidus with the recurrent symptom of facial numbness. The patient underwent a craniotomy due to intractable facial numbness and the surgery was associated with postoperative neurological deficits (a) MRI imaging of T2 at the time of GKRS with radiation volume of 2.4 cc with 12 Gy in 50% line (b) MRI imaging of T2 one year after GKRS with nidus volume of 0.5 cc (c) MRI imaging of T2 weighted 2 years after GKRS with nidus volume of 2.0 cc (d) MRI imaging of T2 at the time of second GKRS with radiation volume of 2.1 cc with 12 Gy in 50% line (e) MRI imaging of T2 9 years after a second GKRS and craniotomy with nidus volume of 1.1cc. Figure S3: Cavernoma treated with GKRS and demonstrating nidus progression and hemorrhage. A 41 year old female suffered facial numbness treated with GKRS and suffered the repeated bleedings (a) MRI imaging of T2, Flair and T1 with contrast at the time of gamma knife treatment with radiation volume of 0.1cc with 12 Gy in 50% line (b) MRI imaging of T2, Flair ,and T1 with contrast three year after GKRS with nidus volume of 0.21 cc (c) CT imaging 5 years after GKRS with intracerebral hemorrhage (d) MRI imaging of T2, Flair ,and T1 with contrast 5 years after GKRS with a nidus volume of 0.023 cc (e) MRI imaging of T2, FLAIR, and T1 with contrast 11 years after GKRS with a nidus volume of 0.021cc. Figure S4: Plot of life quality before and after GKRS. (a) Plot of life quality including SF-36 and KPS before and after GKRS. (b) Plot of life quality of SF-36 and KPS stratified by the etiologies of intracerebral hemorrhage and seizure. *: p<0.05; **:p<0.01. Figure S5: Plots of life quality of SF-36 and KPS in the patients either with or without development of new neurological deficits. **: p<0.01. Table S1: The associated factors contributing to the improvement of physical component. Table S2: The associated factors contributing to improvement in mental component. Table S3: The associated factors contributing to the improvement in KPS.

## Data Availability

All data generated or analyzed during this study are included in this published article and its additional files.

## Abbreviations

GKRS: Gamma Knife Radiosurgery
SRS: Stereotactic radiosurgery
TV: Target volume
Gy: Gray
MRI: Magnetic resonance imaging
Gd: Gadolinium
CM: Intracerebral cavernoma
ICH: Intracranial bleeding
KPS: Karnofsky performance score
**QOL**: Quality of life
PF: Physical function
RP: Role limitation due to a physical problem
BP: Bodily pain
GH: General health
SF: Social functioning
RE: Role limitation due to an emotional problem
MH: Mental health (MH)
