# Supplementary material for: Outcome of intracerebral cavernoma treated by Gamma Knife radiosurgery based on a double-blind assessment of treatment indication
Source: Radiat Oncol. 2021 Aug 28;16:164. doi: 10.1186/s13014-021-01885-4 (PMC8401103; doi:10.1186/s13014-021-01885-4)
Supplement: Supplementary file 1 — Additional file 1. Figure S1: The treatment protocol was presented as a schematic flowchart. Figure S2: Cavernoma treated with nidus progression followed by second GKRS and craniotomy. A 29 year old female suffered facial numbness treated with GKRS and received a second GKRS due to increased volume of nidus with the recurrent symptom of facial numbness. The patient underwent a craniotomy due to intractable facial numbness and the surgery was associated with postoperative neurological deficits (a) MRI imaging of T2 at the time of GKRS with radiation volume of 2.4 cc with 12 Gy in 50% line (b) MRI imaging of T2 one year after GKRS with nidus volume of 0.5 cc (c) MRI imaging of T2 weighted 2 years after GKRS with nidus volume of 2.0 cc (d) MRI imaging of T2 at the time of second GKRS with radiation volume of 2.1 cc with 12 Gy in 50% line (e) MRI imaging of T2 9 years after a second GKRS and craniotomy with nidus volume of 1.1cc. Figure S3: Cavernoma treated with GKRS and demonstrating nidus progression and hemorrhage. A 41 year old female suffered facial numbness treated with GKRS and suffered the repeated bleedings (a) MRI imaging of T2, Flair and T1 with contrast at the time of gamma knife treatment with radiation volume of 0.1cc with 12 Gy in 50% line (b) MRI imaging of T2, Flair ,and T1 with contrast three year after GKRS with nidus volume of 0.21 cc (c) CT imaging 5 years after GKRS with intracerebral hemorrhage (d) MRI imaging of T2, Flair ,and T1 with contrast 5 years after GKRS with a nidus volume of 0.023 cc (e) MRI imaging of T2, FLAIR, and T1 with contrast 11 years after GKRS with a nidus volume of 0.021cc. Figure S4: Plot of life quality before and after GKRS. (a) Plot of life quality including SF-36 and KPS before and after GKRS. (b) Plot of life quality of SF-36 and KPS stratified by the etiologies of intracerebral hemorrhage and seizure. *: p<0.05; **:p<0.01. Figure S5: Plots of life quality of SF-36 and KPS in the patients either with or without develop [file 13014_2021_1885_MOESM1_ESM.pdf]

Supplemental data

Supplemental Figures:

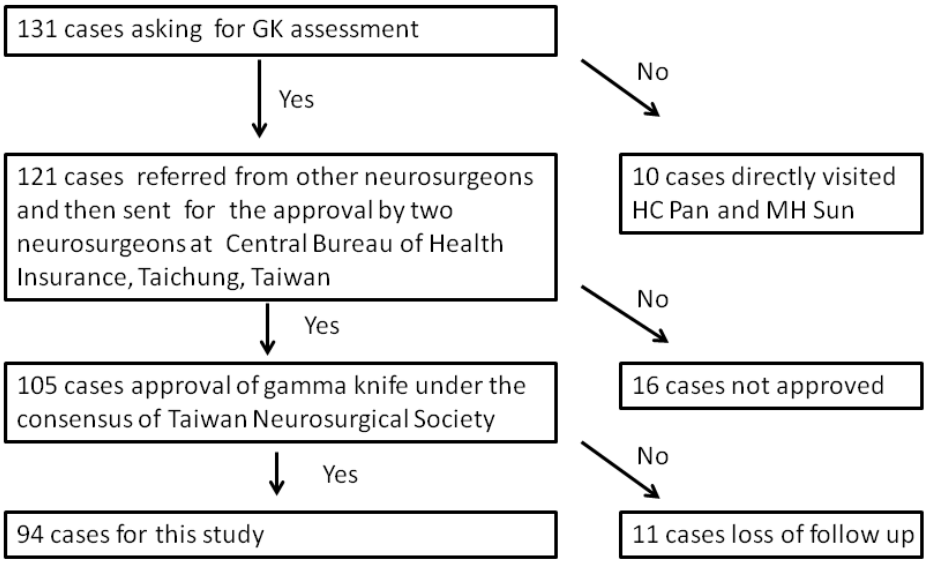

Supplemental Fig. 1: The treatment protocol was presented as a schematic flowchart.

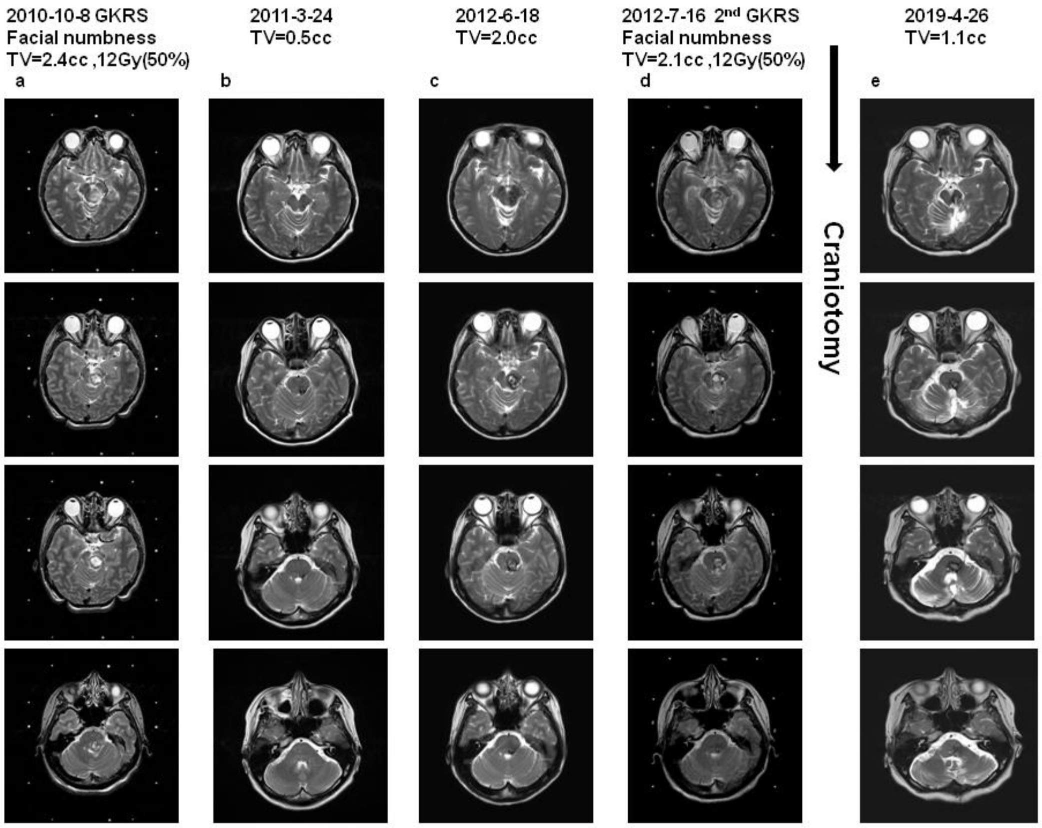

Supplemental Fig. 2: Cavernoma treated with nidus progression followed by second GKRS and craniotomy. A 29 year old female suffered facial numbness treated with GKRS and received a second GKRS due to increased volume of nidus with the recurrent symptom of facial numbness. The patient underwent a craniotomy due to intractable facial numbness and the surgery was associated with postoperative neurological deficits (a) MRI imaging of T2 at the time of GKRS with radiation volume of 2.4 cc with 12 Gy in 50% line (b) MRI imaging of T2 one year after GKRS with nidus volume of 0.5 cc (c) MRI imaging of T2 weighted 2 years after GKRS with nidus volume of 2.0 cc (d) MRI imaging of T2 at the time of second GKRS with radiation volume of 2.1 cc with 12 Gy in 50% line (e) MRI imaging of T2 9 years after a second GKRS and craniotomy with nidus volume of 1.1cc

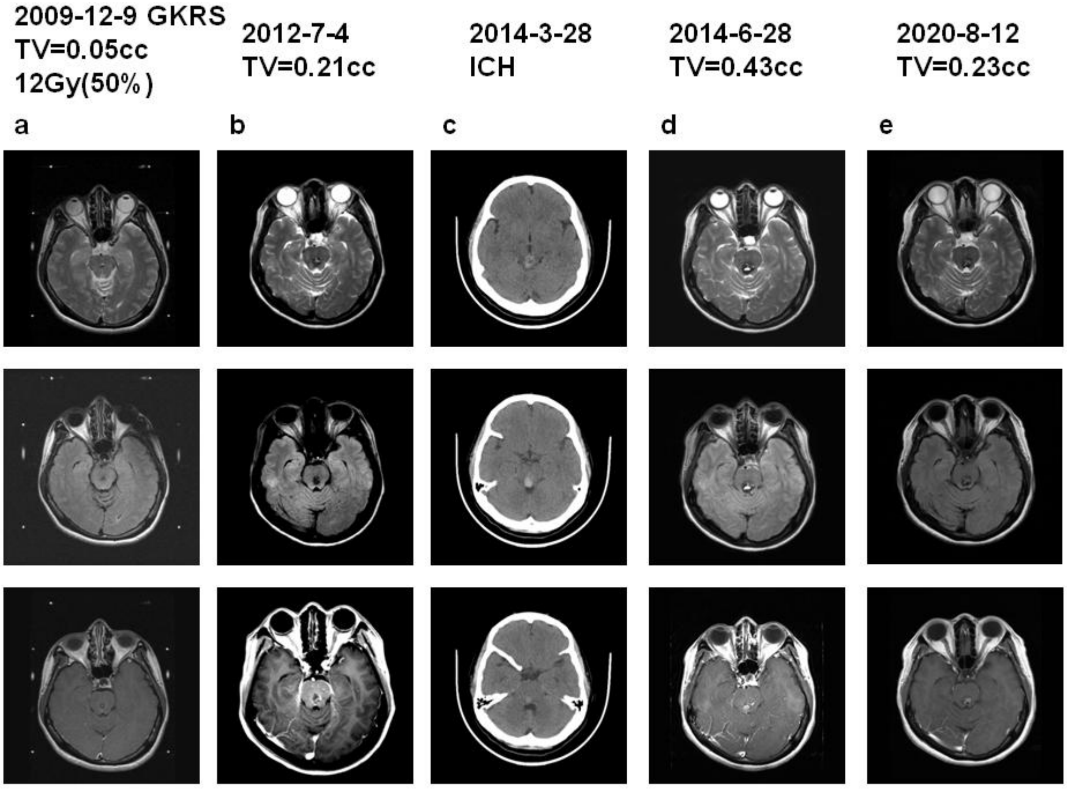

Supplemental Fig. 3: Cavernoma treated with GKRS and demonstrating nidus progression and hemorrhage. A 41 year old female suffered facial numbness treated with GKRS and suffered the repeated bleedings (a) MRI imaging of T2, Flair and T1 with contrast at the time of gamma knife treatment with radiation volume of 0.1cc with 12 Gy in 50% line (b) MRI imaging of T2, Flair ,and T1 with contrast three year after GKRS with nidus volume of 0.21 cc (c) CT imaging 5 years after GKRS with intracerebral hemorrhage (d) MRI imaging of T2, Flair ,and T1 with contrast 5 years after GKRS with a nidus volume of 0.023 cc (e) MRI imaging of T2, FLAIR, and T1 with contrast 11 years after GKRS with a nidus volume of 0.021cc

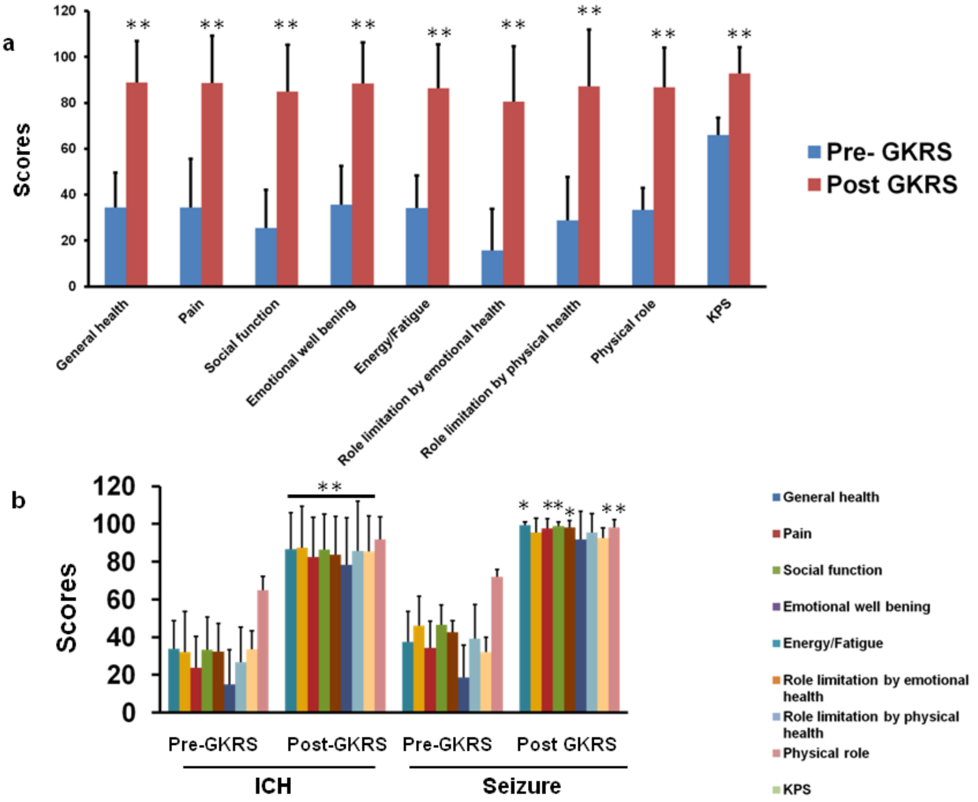

Supplemental Fig. 4: Plot of life quality before and after GKRS. (a) Plot of life quality including SF-36 and KPS before and after GKRS. (b) Plot of life quality of SF-36 and KPS stratified by the etiologies of intracerebral hemorrhage and seizure. *: p<0.05; **:p<0.01

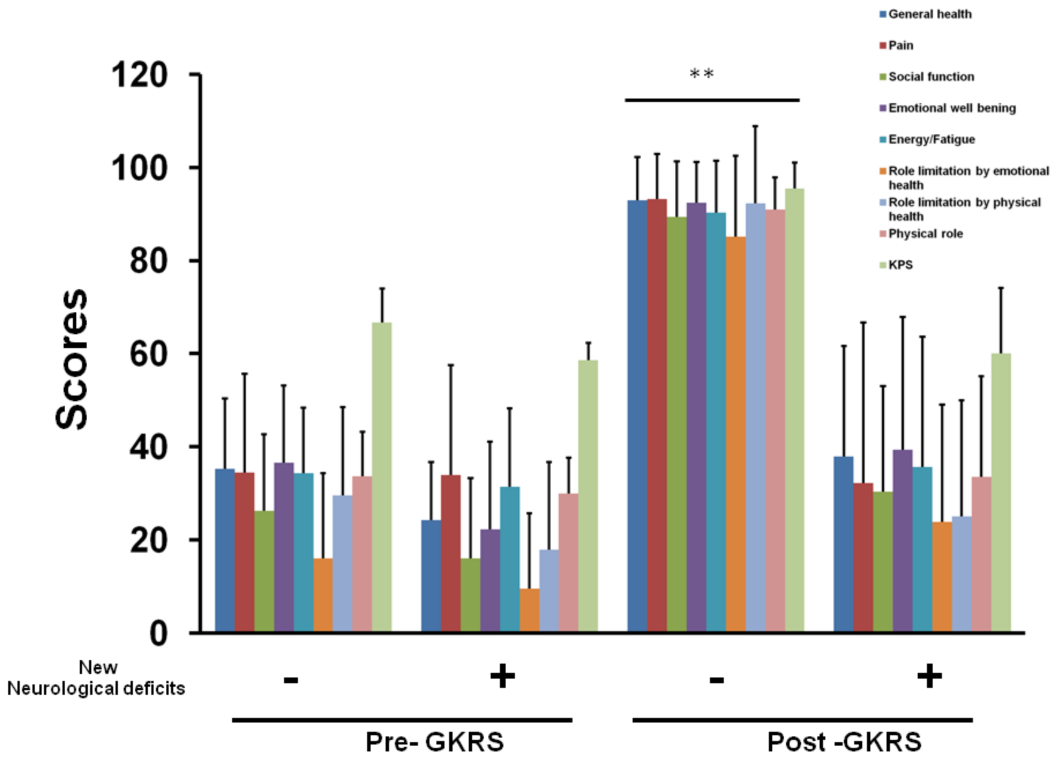

Supplemental Fig. 5: Plots of life quality of SF-36 and KPS in the patients either with or without development of new neurological deficits. **: p<0.01

Supplemental Tables:

Supplemental table 1: The associated factors contributing to the improvement of physical component

	Simple model		Multiple model	
	OR (95%CI)	*p* value	OR (95%CI)	*p* value
Age	1.06 (0.99-1.14)	0.070		
Sex				
Female	ref.			
Male	2.98 (0.32-27.75)	0.337		
Time from diagnosis to GK (months)	0.98 (0.84-1.14)	0.769		
Neurological deficits	0.50 (0.08-3.12)	0.456		
TV(cc)	1.48 (0.59-3.67)	0.401		
Margin dose (Gy)	0.73 (0.34-1.54)	0.406		
Multiple Lesions	1.74 (0.19-16.32)	0.627		
Venous abnormality	0.04 (0.004-0.34)	0.004	0.14 (0.01-3.04)	0.210
Post GK volume				
decrease + stable	ref.		ref.	
increase	0.003 (0.0001-0.05)	<0.001	0.01 (0.0003-0.15)	0.002

Logistic regression. *p<0.05, **p<0.01.

OR: Odds Ratio

Supplemental table 2:The associated factors contributing to improvement in mental component

	Simple model		Multiple model	
	OR (95%CI)	*p* value	OR (95%CI)	*p* value
Age	1.08 (0.99-1.17)	0.070		
Sex				
Female	ref.			
Male	2.19 (0.22-21.90)	0.504		
Time from diagnosis to GKRS (months)	0.95 (0.82-1.11)	0.543		
Neurological deficits	0.76 (0.10-5.67)	0.793		
TV(cc)	1.28 (0.58-2.80)	0.537		
Margin dose (Gy)	0.59 (0.25-1.38)	0.221		
Multiple Lesions	1.29 (0.13-12.92)	0.831		
Venous abnormality	0.05 (0.005-0.53)	0.013	0.26 (0.01-5.85)	0.400
Post GKRS volume				
decrease + stable	ref.		ref.	
increase	0.01 (0.001-0.11)	<0.001	0.02 (0.001-0.35)	0.008

Logistic regression. *$p<0.05$, **$p<0.01$.

OR: Odds Ratio

Supplemental Table 3: The associated factors contributing to the improvement in KPS

	Simple model		Multiple model	
	OR (95%CI)	*p* value	OR (95%CI)	*p* value
Age	1.04 (0.98-1.11)	0.191		
Sex				
Female	ref.			
Male	2.98 (0.32-27.75)	0.337		
Time from diagnosis to GKRS (months)	1.16 (0.81-1.65)	0.417		
Neurological deficits	1.17 (0.19-7.35)	0.867		
TV(cc)	1.12 (0.70-1.77)	0.642		
Margin dose (Gy)	0.63 (0.29-1.35)	0.231		
Multiple Lesions	1.74 (0.19-16.32)	0.627		
Location				
brain stem + deep seated	ref.			
subcortical	2.85 (0.31-26.51)	0.358		
Venous abnormality	0.04 (0.004-0.34)	0.004	0.14 (0.01-3.04)	0.210
Post GKRS volume				
decrease + stable	ref.		ref.	
increase	0.003 (0.0001-0.05)	<0.001	0.01 (0.0003-0.15)	0.002

Logistic regression. *$p<0.05$, **$p<0.01$.

OR: Odds Ratio
